# Variability in the Precision of Children’s Spatial Working Memory

**DOI:** 10.3390/jintelligence6010008

**Published:** 2018-02-28

**Authors:** Elena M. Galeano Weber, Judith Dirk, Florian Schmiedek

**Affiliations:** 1 German Institute for International Educational Research (DIPF), Frankfurt am Main, Frankfurt 60486, Germany; schmiedek@dipf.de (J.D.); dirk@dipf.de (F.S.); 2 Individual Development and Adaptive Education (IDeA) Center, Frankfurt 60486, Germany

**Keywords:** working memory updating, spatial precision, intra-individual variability, cognitive development, micro-longitudinal design, ambulatory assessment, hierarchical modeling

## Abstract

Cognitive modeling studies in adults have established that visual working memory (WM) capacity depends on the representational precision, as well as its variability from moment to moment. By contrast, visuospatial WM performance in children has been typically indexed by response accuracy—a binary measure that provides less information about precision with which items are stored. Here, we aimed at identifying whether and how children’s WM performance depends on the spatial precision and its variability over time in real-world contexts. Using smartphones, 110 Grade 3 and Grade 4 students performed a spatial WM updating task three times a day in school and at home for four weeks. Measures of spatial precision (i.e., Euclidean distance between presented and reported location) were used for hierarchical modeling to estimate variability of spatial precision across different time scales. Results demonstrated considerable within-person variability in spatial precision across items within trials, from trial to trial and from occasion to occasion within days and from day to day. In particular, item-to-item variability was systematically increased with memory load and lowered with higher grade. Further, children with higher precision variability across items scored lower in measures of fluid intelligence. These findings emphasize the important role of transient changes in spatial precision for the development of WM.

## 1. Introduction

Working memory (WM) refers to the temporal storage and manipulation of sensory information online [1]. It is considered to be a core cognitive process that is severely limited in capacity [2,3,4,5]. WM for visuospatial information supports mental arithmetic [6,7,8], spatial thinking [9,10] and fluid intelligence [11,12,13]. Such higher cognitive functions are implicated to be essential for learning and development [14] but the specific factors that contribute to visual WM limitations in children are still not clear. Here, we aimed at identifying a cognitive component, spatial precision, that contributes to developmental changes and limitations in children’s visuospatial WM updating performance in natural everyday life contexts.

### 1.1. Models of Visual Working Memory Capacity

Visual WM capacity can be measured by varying the number of objects that have to be remembered [2,3]. Fixed capacity or ‘slot’ models of visual WM suggest a limit of three to four storage slots, one of each object held in WM [2]. It has been criticized that slot models do not account for the presence of internal noise in memory which increases with increasing load [3,4]. Thus, WM may not store a limited number of discrete representations but rather consists of a flexible resource: the more of this resource is allocated to an item, the less noise is present in its representation and the more reliable is the recall of that item [4,15]. Responses during recall could be corrupted by many sources of noise including sensory, perceptual, mnemonic, and/or motor noise [3,4]. More recently, cognitive modeling studies in human adults have established that visual WM capacity is constrained by the precision with which items are stored [15,16]. In particular, visual WM capacity has been formalized through distinct components such as the probability to guess at random [2,16], the probability of misremembering features of non-target items or binding errors [17,18], the precision of memory representation and its decline with load [15,16,19] and the variability of precision [20,21], rather than through a fixed limit to the number of objects that can be stored [3]. Importantly, most recent versions of flexible resource models suggest that WM resource is not equally but variably distributed across items and trials. Thus, precision itself is allowed to vary over time, across objects and across conditions within individuals [20,21]. To test these assumptions, different models were compared and fit to errors in recall (i.e., the difference between the participant’s estimate and the true stimulus value) [22] measured with delayed-estimation tasks [19]. Results showed that model variants with a combination of several components, including a variable precision parameter, were most successful and outperformed models that did not consider variability in precision [22].

### 1.2. Variability in Working Memory Performance

The comparison of visual WM models revealed that human adults show substantial variability in WM precision across trials within a testing session, which is implicated to strongly contribute to capacity limitations [20,21,22]. Based on findings that visual cues during stimulus encoding can increase WM precision, shifts of attention could constitute a possible source of variability in precision [23]. By contrary, precision variability could result from random fluctuations in attention, when multiple items have to be remembered [21]. Further, variability in WM performance has been linked to dopamine activity [24], whereby dopaminergic stimulation in the prefrontal cortex can modulate visuospatial WM [25,26]. In addition to such rapid trial-to-trial variability in WM separated by milliseconds or seconds [20,21,22,27], intra-individual WM variability has also been reported for slower time scales, such as across sessions within days or even from day to day [28,29,30,31,32]. In these studies, memory span, updating, or delayed spatial recognition tasks were repeatedly administered to younger and older adults embedded in intensive microlongitudinal designs [32]. In this way, it has been demonstrated that WM fluctuations from day to day are related to fluctuations in motivation [33], mood states [29] and affect [34]. Moreover, trial-to-trial variability in measures of reaction time was shown to follow a u-shaped function across the lifespan where children and older adults were more variable in their WM performance than younger adults [35]. When evaluating day-to-day variability and measures of WM accuracy, however, older adults showed lower variability compared to younger adults and thus more stable performance [31]. Together, these findings highlight the importance to consider different time scales and different cognitive measures when evaluating intra-individual variability in WM functioning [31].

### 1.3. Development of Children’s Working Memory

Studies on children’s WM variability are scarce [35,36,37,38,39]. Moreover, thus far, only a few studies have investigated the contribution of children’s WM precision for age- and load-related performance changes [40,41,42,43,44,45]. For example, Burnett Heyes and colleagues (2012) observed developmental increases in visual WM precision (the reciprocal of the standard deviation of a continuous response distribution) in 7 to 13 years old boys [40]. In comparison, Sarigiannidis and colleagues (2016) found reduced guessing behavior (i.e., height parameter of a discrete probability distribution) in older (aged 10–12) compared to younger (aged 7–9) children, rather than improvements in precision [42]. In addition to these mixed results, so far, it is not clear how WM precision and in particular the moment-to-moment variability of this precision changes across development. In a recent study from our own lab, fluctuations in children’s WM updating performance were assessed over a period of four weeks in the school context. Results revealed that WM accuracy systematically fluctuates across and within days and across moments. Here, children strongly differed in their amount of reliable variability in accuracy at these different time scales whereby third graders were more variable within days than fourth graders [37].

### 1.4. Research Questions and Approach

Taken together, the existing research emphasizes a critical role of distinct WM components for visual WM capacity limitations in adults and children (e.g., [22,37,40,42]). Beyond temporally holding sensory information in visual WM, spatial WM updating requires children to constantly update the locations of multiple items. A precise representation of each item’s location may be beneficial to successfully solve the task. However, updating performance has been typically indexed by response accuracy–a binary measure that only provides information whether children have correctly recalled the item or not. In addition, while environmental contexts and life conditions doubtlessly affect cognitive development [46], limitations in children’s WM precision have been typically studied in the laboratory. Thus, an ecologically valid assessment of children’s WM precision and its variability over time is still missing but may reveal further insights into cognitive processes in everyday contexts. To investigate such processes, microgenetic approaches and intensive longitudinal designs allow the assessment of rapidly changing processes with high density of observations within a given period of time [47]. In this regard, intensive longitudinal designs in combination with ambulatory assessment has proven to be a fruitful approach to measure WM updating fluctuations at different time scales in children’s daily lives [37,38]. By adopting cognitive tasks for mobile devices, dynamics of behavior and developmental processes can be examined in a reliable and feasible way [48]. Based on these considerations, here we aimed at further identifying and comparing distinct components that limit visuospatial WM updating in children’s natural environment. Because WM has been demonstrated to be an important predictor of academic attainment (e.g., [14,37]) and variance in WM performance related to age and years of schooling is expected to overlap considerably in the present study, we focused on grade differences instead of age-related changes. In particular, we measured spatial precision and estimated variability in spatial precision at different time scales in Grade 3 and Grade 4 students who performed a sequential visuospatial updating task three times daily over a period of four weeks using smartphones.

By taking into account recent developmental findings on visual and spatial WM capacity [40,41,42,43,44], we assumed that spatial precision declines as load on WM updating increases (i.e., from a memory load of two to a load of three) and that spatial precision increases with level of education (Grade 3 vs. 4). Following recent findings of variability in updating accuracy [37] and cognitive modeling of precision in adults [20,21], we tested whether spatial precision of WM updating systematically varies within children by considering different time scales (i.e., items, trials, occasions and days), effects of load and level of education. Specifically, by considering recent theoretical considerations of variable precision models [20,21], we assumed that the amount of rapid fluctuations in spatial precision may increase with load due to an increased level of children’s internal noise. Finally, we explored individual differences in fluid intelligence and its relation to different variance components of spatial precision.

## 2. Materials and Methods

The present study is based on data from the FLUX project (‘Assessment of Cognitive Performance FLUctuations in the School ConteXt’) of the Individual Development and Adaptive Education (IDeA) Center in Frankfurt, Germany. The project followed an intensive microlongitudinal design with four daily assessments over a period of four weeks (28 or 31 consecutive days including weekend days) embedded in a pre- and posttest protocol. Within this project, cognitive performance [37,38], motivation, affect [49], sleep [38,50] and physical activity [51], amongst other variables, were assessed on a daily basis via smartphones (Dell Streak 5, with Android 2.2 operation system). In this study, we considered daily measures from a visuospatial WM updating task and background measures from a pretest session such as demographic variables, fluid intelligence (i.e., *CFT 20-R*, [52]) and school achievement including a mathematics test (i.e., *DEMAT*, [53] and reading comprehension test (i.e., *ELFE*, [54]). Pretest assessment took place in the classroom in groups of up to 20 students and started one week before the longitudinal study phase (see [37], for a description of study protocol). 

### 2.1. Participants

Participants were 110 third- and fourth-graders aged between 8 and 11 years (65 boys, *M* = 9.88 years, *SD* = 0.61, *range* = 8.1). Fifty children of the sample were enrolled in Grade 3 (26 boys, *M* = 9.40 years, *SD* = 0.46, *range* = 2.3) and 60 in Grade 4 (39 boys, *M* = 10.27 years, *SD* = 0.39, *range* = 1.6). Children’s fluid intelligence was in an average range with *M* = 106.9 (*SD* = 12.8) and *M* = 109.3 (*SD* = 17.3) for Grade 3 and Grade 4 students, respectively. Grade 4 students significantly differed in fluid intelligence from Grade 3 students (*CFT 20-R* raw scores: Grade 4: *M* = 33.59 (*SD* = 7.31) > Grade 3: *M* = 29.84 (*SD* = 5.37), *t* = 3.05, *df* = 103.12, *p* < 0.05). They were recruited from seven classes in one public elementary school in an average urban neighborhood in Frankfurt am Main, Germany. Participation was voluntary and could be canceled anytime without giving reasons. The children received a gift certificate or money for participation. Informed consent was obtained in accordance with a protocol approved by the local ethics review board. 

### 2.2. Procedure

Children completed a visuospatial WM updating task on three daily sessions over a period of four weeks. WM performance was tested in the morning during class (Occasion 1), at noon at the end of school (Occasion 2) and in the afternoon (Occasion 3). School sessions were scheduled to fixed times for all children, afternoon sessions could be scheduled individually within a time window of ±2 h and sessions were available up to 60 min. Within each occasion, the spatial updating session followed a numerical updating session in which children had to remember and update numbers in WM (cf. [37]). The spatial WM updating task comprised eight trials per session. Each session started with four trials of memory Load 2 (=2 items), followed by four trials of memory Load 3 (=3 items). Children’s responses were consecutively measured for each item held and updated in WM. In each trial, two or three responses could be obtained for a manipulation of memory Load 2 or 3, respectively. Thus, in one session (occasion), children were able to give 20 responses in total. In the course of study period, a maximum of 91 sessions (Grade 3) or 84 sessions (Grade 4) could be completed. Thus, in total, a maximum of 364/336 responses (Grade 3/Grade 4) to the first, second, or third item within trials could be collected for each child.

### 2.3. Spatial Working Memory Updating Task

**Figure 1:**
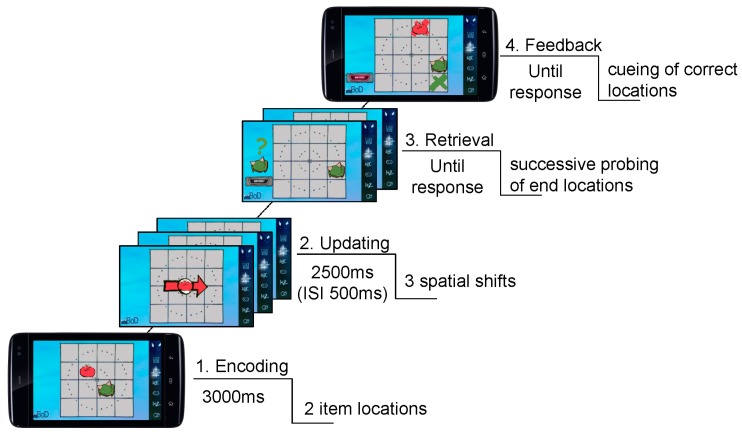
Visuospatial working memory updating task (example showing Load 2). Children had to encode, hold and update the locations of two or three items in visual WM. After updating operations (i.e., sequential mental shifts within a 4 × 4 spatial grid), children were prompted to retrieve the updated locations. Responses were consecutively given to each item by touching on the remembered location (cf. [37]).

Children had to memorize and update locations of differentially colored and shaped cartoon creatures (=items) presented in a 4 × 4 grid. During the encoding phase, two or three items were presented simultaneously at different locations in the grid for 3000 ms. After an inter-stimulus-interval (ISI) of 500 ms, three or four updating cues were presented for Load 2 and Load 3 conditions, respectively. Updating cues were shown in the center of the grid and were presented sequentially. Each cue was shown for 2500 ms with an ISI of 500 ms. Each item of the sample display was assigned to one respective cue. Cues were cartoon arrows that matched the item’s colors where the respective item was placed at the center of an arrow. The direction of the arrow prompted children to mentally shift the spatial position of the respective item to the adjacent location in the grid (= updating operation). Directions of arrows were horizontal (left, right), vertical (upper, below), or diagonal. No item’s position could be updated twice in a row. Intermediate and end positions were never doubly assigned. After updating, children had to retrieve updated positions for each item within a trial. They responded by consecutively touching the remembered item location. Target locations were indicated by the corresponding item and a question mark sign that were shown left to the grid. A feedback followed by showing color-coded crosses at correct locations after the final response was given (Figure 1) (cf. [37]; task was adopted from [55]).

### 2.4. Data Analysis

**Figure 2:**
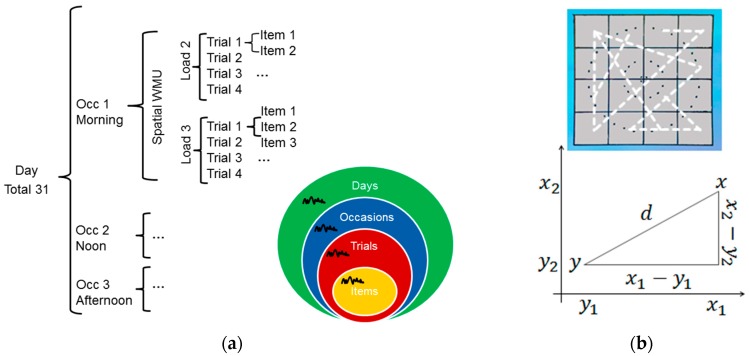
Hierarchical data structure and spatial precision: (**a**) Data were inherently structured by repeated measures at four different levels of time scale (i.e., Items, Trials, Occasions and Days) given the microlongitudinal study design; (**b**) Spatial precision was formalized in terms of the Euclidean Distance δ between presented (*y*) and reported location (*x*) within a 4 × 4 space grid (cf. upper panel). Dashed white lines represent possible distances in the space grid ranging from δ = 0 to a maximum of δ = 4.24.

Behavioral data were analyzed using the *lme4* package [56] as well as core packages in R-statistics (https://www.r-project.org, R Core Team, 2016). Given the intensive longitudinal design, observations were inherently structured by repeated measures across items (Level 1) that were nested within trials (Level 2), measures across trials, in turn, were nested within occasions (Level 3) and assessment at occasions were nested within days (Level 4) (Figure 2a). Item responses were measured in terms of continuous spatial precision (i.e., Euclidean distance) in addition to discrete accuracy (i.e., correct vs. incorrect) (cf. Section 2.5). The hierarchical data structure allowed for decomposing the four different variance components of spatial precision for each individual (cf. Section 2.5).

With an intensive longitudinal study protocol, missing data were expected. Here, on average across load conditions and Grades, 67% of the maximum possible visual spatial WM updating data were available. Missing data resulted from, for example, illness, exams, technical problems such as empty batteries, or smartphones left at home. Based on available data, the average total number of responses from Grade 3 students was 232.98 (*SD* = 86.57) and 232.74 (*SD* = 86.58) for first and second items in Load 2 trials and 230.96 (*SD* = 87.97), 230.76 (*SD* = 87.94) and 230.58 (*SD* = 87.99) for first, second and third items of Load 3 trials, respectively. Grade 4 students responded on average 239.82 (*SD* = 67.18) and 239.72 (*SD* = 67.20) times to the first and second item in Load 2 trials and 238.75 (*SD* = 67.97), 238.63 (*SD* = 67.99) and 238.58 (*SD* = 67.98) to first, second and third items in Load 3 trials, respectively. Sufficient data for hierarchical modeling analysis and sufficiently reliable estimation of individual variance components were assumed for children with more than 20 days (cf. [37]). Thus, load effects on variability of spatial precision and individual differences in variance components were assessed based on data of 83 children for whom sufficient observations were available to estimate variance components at different timescales. All other analyses were based on data from the entire sample of 110 children.

### 2.5. Scoring Behavioral Performance

*Spatial precision* was formalized as Euclidean Distance between response location and original location for each item (cf. [57]). The Euclidean Distance is defined as the distance between two points in space that corresponds to the length of a straight line drawn between them, where the distance δ from x to y or y to x is given by the following Pythagorean formula:

(1) $$\delta \left(x,y\right)=\sqrt{{\left(x_{1}-y_{1}\right)}^{2}+{\left(x_{2}-y_{2}\right)}^{2}},$$

Here, we assume that a higher δ may reflect more dissimilar representations between presented and reported item location, which may result from less spatially precise memory representations due to increased memory noise (e.g., [15]). The Euclidean metric works well for two-dimensional spaces and reflects a more sensitive measure of spatial recall precision as compared to the number of cells as a distance measure. For example, placing an item in a cell that touches the correct cell diagonally (δ = 1.41) is considered a somewhat larger error than placing it in a cell that touches the correct cell horizontally or vertically (δ = 1). The metric space of δ was a 4 × 4 cell grid where one cell reflects one of 16 different item locations. Specifically, we computed the square root of the sum of the squares of the difference between all corresponding values within a 4 × 4 matrix (e.g., *x*(1,2) and *y*(2,3)) by using the *dist* function in R. This resulted in nine distinct δ values ranging from δ = 0 to a maximum of δ = 4.24 and 120×(15×15 − 1)/2 possible pairs of presented and reported location (cf. Figure 2b).

For *response accuracy*, a given response was assigned a value of 1 for correct responses (when the correct location of the target item was chosen) and a value of 0 for erroneous responses (when any other location except the correct location was chosen).

For data analysis across trials (Level 2), spatial precision and accuracy scores were obtained by averaging across responses for each item within trials. For analysis at the occasion level (Level 3), the mean spatial precision and mean accuracy of all responses of the four trials per session and load condition was obtained. To test whether performance in mean spatial precision differs between morning, noon and afternoon sessions, we conducted paired t-tests between levels of *Occasion* (i.e., *morning, noon, afternoon*) separately for each load condition.

*Variance components of spatial precision.* Separately for each child and each load condition, a multilevel model was set up with the dependent variable being spatial precision, that is, the Euclidean Distance between presented and reported location for each item. The model’s intercept parameter is composed of a fixed and random effects, the slope parameter has only a fixed effect. In particular, the model allowed for random intercepts of each time scale that were nested within each other. Running trial number was included as a continuous predictor and modeled as fixed effect to take into account individual longer-term trends. This general model resulted in four different variance components of spatial precision: A variance component of day-to-day variability across the n daily occasions (σ^2^_Days_), a component of occasion-to-occasion variability across the n trials within occasions divided by the number of occasions within days (σ^2^_Occasion_), trial-to-trial variance across the n item-responses within trials divided by the number of trials within days (σ^2^_Trial_) and the variance component of item-to-item variability, including also error variance, divided by the number of responses within days (σ^2^_Item_).

To test whether mean spatial precision and variability of spatial precision across different time scales change as a function of WM load (i.e., Load 3 vs. 2), we conducted paired t-tests separately for each performance component. Further, we assessed individual differences in children’s estimated variance components of spatial precision at different time scales. We tested for differences in spatial precision performance between school classes using independent t-tests. Finally, we assessed the relationship between mean and variability of spatial precision and measures of fluid intelligence (i.e., *CFT 20-R* raw scores) and school achievement (i.e., *ELFE* and *DEMAT* raw scores) using correlation and hierarchical regression analyses. These analyses were based on subsamples of 82, 79, or 73 children (i.e., for *CFT*, *DEMAT*, *ELFE*, respectively) for whom scores and sufficient data for estimating variance components of spatial precision were available. Results were considered to be significant when *p* < 0.05 by applying a Bonferroni correction to take into account multiple comparisons. 

## 3. Results

### 3.1. Relationship between Mean Spatial Precision and Mean Response Accuracy

**Figure 3:**
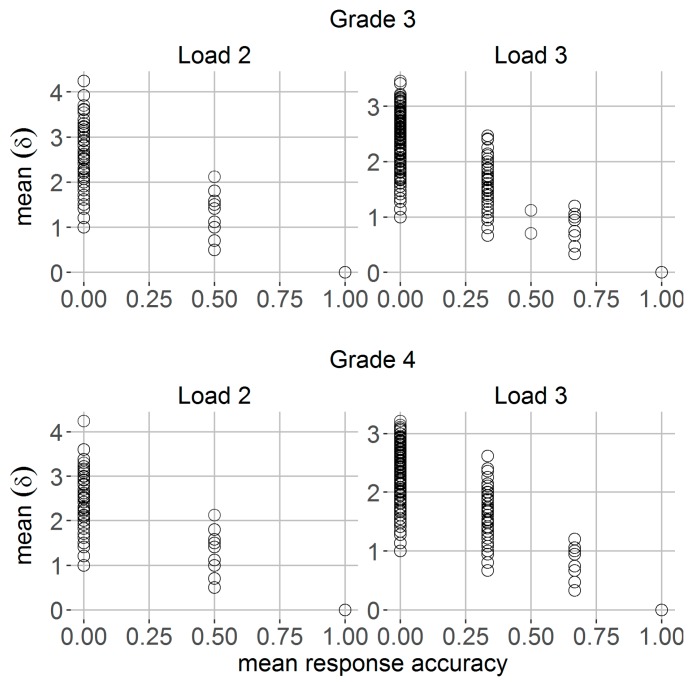
Trial-to-trial mean spatial precision (i.e., Euclidean distance δ) (y-axis) as a function of mean response accuracy (x-axis) for Grade 3 (first row) and Grade 4 students (second row) and separately for loads two and three.

For each trial, mean behavioral performance scores were computed by averaging across data from item-to-item responses. Note that mean response accuracy corresponds to the probability of remembering the correct target location, while mean spatial precision corresponds to participant’s recall precision of spatial location in terms of the mean spatial distance δ between correct and reported location. A mean Euclidean distance δ of 0 corresponds to memory representations with perfect spatial precisions, while a mean δ of 4.24 reflects most imperfect or imprecise spatial representations within trial (which could result from a true location in one of the corners of the grid being remembered as the diagonally opposite corner). Trial-to-trial mean response accuracy ranged from 0 (i.e., incorrect remembered locations) to 1 (i.e., correct remembered locations). Figure 3 shows the relationship between these two parameters and indicates that trial-to-trial mean spatial precision δ varies widely when there was in fact no variation for mean response accuracy. For both grades of school, this variation in spatial precision was most pronounced for erroneous responses (i.e., mean response accuracy = 0) (Figure 3).

### 3.2. Daily Measures of Spatial Precision

To further assess the role of daily spatial precision in WM updating, we compared mean performance at different occasions, that is, average Euclidean distances δ in morning, noon and afternoon sessions within days in Grade 3 and Grade 4 students. Descriptive results demonstrated best performance in terms of lowest mean δ for Grade 4 students and Load 2 condition during morning sessions (*M* = 0.30, *SD* = 0.23), while lowest spatial precision was observed for Grade 3 students and Load 3 during noon (*M* = 1.11, *SD* = 0.39; cf. Table S1). Children showed highest mean spatial precision during sessions in the morning, while lowest performance was observed during noon sessions (Load 2/3: *t* ≤ −8.33, *df* = 109, *p* < 0.05). Further, results demonstrated reduced spatial precision in noon compared to afternoon sessions (Load 2/3: *t* ≥ 4.29, *df* = 109, *p* < 0.05) and higher spatial precision during morning than afternoon (Load 2/3: *t* ≤ −3.36, *df* = 109, *p* < 0.05).

### 3.3. Variability in Spatial Precision

**Figure 4:**
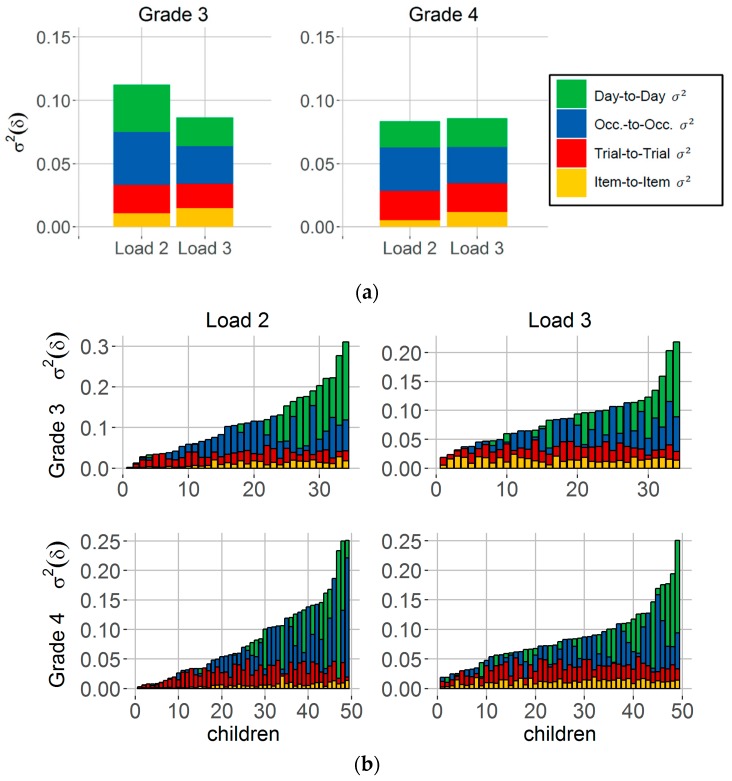
Variance components of spatial precision. (**a**) The size of each bar reflects the total amount of children’s averaged observed day-to-day variability separately for Grade 3 and Grade 4 students and load conditions (i.e., Load 2 and 3). This variability is decomposed into four different variance components that were estimated for each individual in each load condition. Variance components reflect variability of spatial precision from item-to-item (yellow), trial-to-trial (red), across occasions (Occ.; blue) and true day-to-day variability (green); (**b**) Each bar corresponds to children’s estimated item-to-item, trial-to-trial, occasion-to-occasion and day-to-day variance component of observed variability across days. Bars are ordered by their total size (i.e., variance of average performance across days) from very low (left) to very high (right) variability. *N* = 34 (Grade 3), *n* = 49 (Grade 4).

For each child and load condition, we estimated variance components using hierarchical modeling to examine systematic within-person variability of spatial precision across different time scales. Figure 4a shows the children’s average estimated variance components σ^2^(δ) separately for school classes. The total size of each bar corresponds to the average amount of observed variability of spatial precision across days (i.e., the variance of mean spatial precision performance from day-to-day). This variability is decomposed into four variance components reflecting the contribution of item-to-item variability (yellow), trial-to-trial variability (red), occasion-to-occasion variability (blue) and true day-to-day variability (green) to observed day-to-day variability. Figure 4a shows that, on average across children, each variance component contributed to the observed total amount of variability across days within grades and load conditions (cf. Figure 4a). For each time scale and load condition, estimated spatial precision variance component was significantly different from zero within children from Grade 3 (Load 2, all: *t* ≥ 3.76, *df* = 33, Load 3: *t* ≥ 4.02, *df* =, *p <* 0.05) and Grade 4 (Load 2: *t* ≥ 3.41, *df* = 48, Load 3: *t* ≥ 4.39, *df* = 48, *p <* 0.05). Detailed summary statistics for each variance component can be found in Table S2 (see Online Supplement).

#### 3.3.1. Effects of Working Memory Load on Mean and Variability of Spatial Precision

As expected, results showed a significant increase in mean Euclidean distance δ with increased load in Grade 3 students (Load 2: *M* = 0.66, *SD* = 0.4, Load 3: *M* = 1.02, *SD* = 0.39; *t* = −17.31, *df* = 49, *p <* 0.05) and Grade 4 students (Load 2: *M* = 0.37, *SD* = 0.26, Load 3: *M* = 0.75, *SD* = 0.34; *t* = −14.66, *df* = 59, *p <* 0.05), suggesting that children showed lower overall spatial precision as load on WM increased.

For the variability in spatial precision, the item-to-item variance component significantly increased with load in children from Grade 3 (Load 2: *M* = 0.011, *SD* = 0.007, Load 3: *M* = 0.014, *SD* = 0.005; *t* = −5.95, *df* = 33, *p* < 0.05) as well as in children from Grade 4 (Load 2: *M* = 0.005, *SD* = 0.004, Load 3: *M* = 0.012, *SD* = 0.004; *t* = −12.27, *df* = 48, *p* < 0.05) (cf. Figure 4a, yellow bars). No differences between load conditions were found for spatial precision variability from trial-to-trial (Grade 3: *t* = 1.58, *df* = 33, *p* = 0.12; Grade 4: *t* = −0.01, *df* = 48, *p* ≥ 0.99), occasion-to-occasion (Grade 3: *t* = 2.16, *df* = 33, *p* ≥ 0.04; Grade 4: *t* = 1.45, *df* = 48, *p* ≥ 0.15), or for true day-to-day variation in spatial precision (Grade 3: *t* = 1.49, *df* = 33, *p* ≥ 0.15; Grade 4: *t* = −0.33, *df* = 48, *p* ≥ 0.74) (see also Table S2, Online Supplement). These effects cannot be attributed to different trends of learning between the two load conditions, as we took into account individual longer-term trends separately for each child and load condition (cf. *Materials and Methods*, subsection *Variance components of spatial precision*). Thus, load-related differences in children’s updating performance can only be observed for the fast item-to-item changes in spatial precision performance within trials but not for the slower variations across trials, occasions, or days.

#### 3.3.2. Individual Differences in Mean and Variability of Spatial Precision

Further, we examined whether Grade 3 students differ from Grade 4 students in their amounts of estimated variance components of spatial precision and to what degree individual spatial precision variability differs between time scales within each grade. Figure 4b summarizes individual differences in estimated variance components of spatial precision. Here, each bar refers to one child and the total size of the bars corresponds to the variance of average performance across days (i.e., observed day-to-day variability) for each child. Bars on the right at each panel correspond to the children who showed highest observed day-to-day variability of spatial precision. Descriptive results indicate that children considerably differ in their individual amount of estimated variance components at different time scales. For example, there are children who varied in spatial precision across all considered time scales where variation was most pronounced from day-to-day in these children. In contrast, there are also children who showed almost no variation across days but substantial variability in spatial precision across items, trials and/or occasions (cf. Figure 4b). In comparison to Grade 3, Grade 4 students showed significantly less item-to-item variability of spatial precision for memory Load 2 (*t* = 4.02, *df* = 48.6, *p <* 0.05) and Load 3 condition (*t* = 2.90, *df* = 67.3, *p <* 0.05). No differences between grades were found for trial-to-trial (Load 2/3: *p* ≥ 0.07), occasion-to-occasion (Load 2/3: *p* ≥ 0.39), or day-to-day variability (Load 2/3: *p* ≥ 0.16). For the overall mean spatial precision, we observed improved performance (i.e., lower mean δ) in Grade 4 students compared to Grade 3 students for Load 2 (Grade 3: *M* = 0.66, *SD* = 0.4, Grade 4: *M* = 0.37, *SD* = 0.26; *t* = 4.44, *df* = 79.57, *p <* 0.05) and Load 3 (Grade 3: *M* = 1.02, *SD* = 0.39, Grade 4: *M* = 0.75, *SD* = 0.34; *t* = 3.89, *df* = 97.94, *p <* 0.05).

#### 3.3.3. Relationship between Spatial Precision Components, Fluid Intelligence and School Achievement

**Figure 5:**
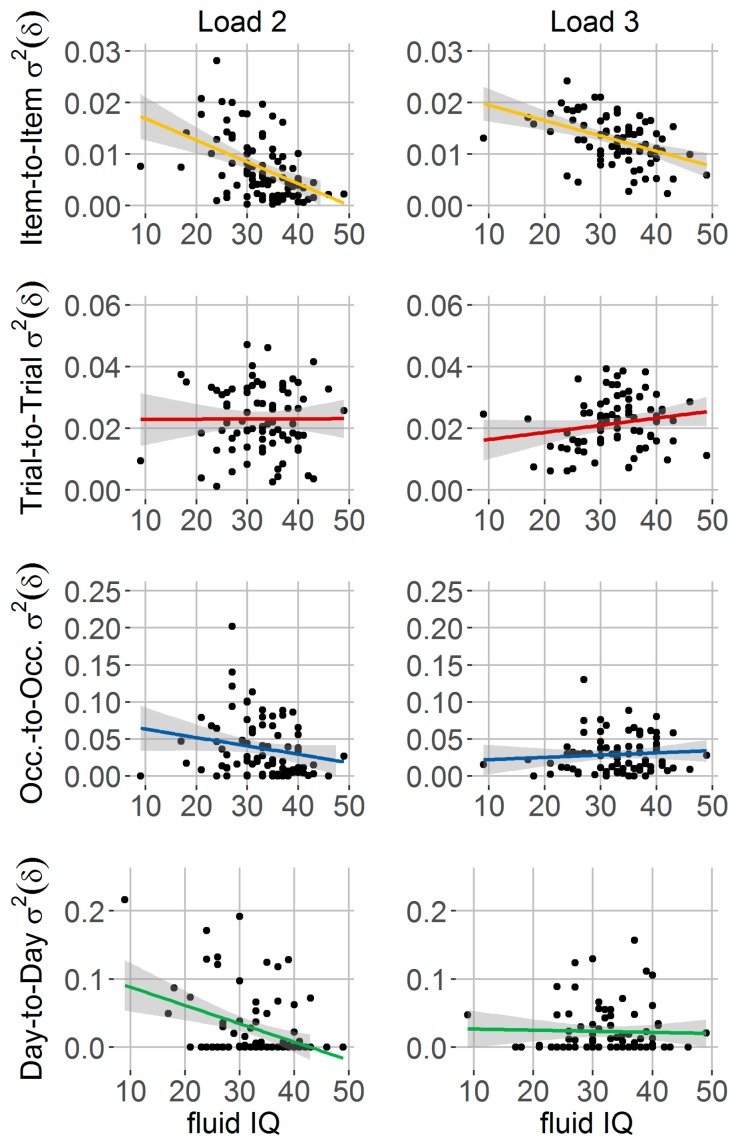
Relationship between fluid intelligence and variability of spatial precision. Children (*n* = 82) with higher scores of fluid intelligence measures (i.e., CFT 20-R raw scores) (x-axis) showed lower item-to-item variability of spatial precision (cf. first row, Load 2/3: *p* < 0.05). No such relationship was found for variability of spatial precision across trials (second row), occasions (third row), or days (fourth row).

Firstly, we assessed the relationship between fluid intelligence (i.e., *CFT-20-R* raw scores) and spatial precision components. For mean spatial precision, results demonstrated that children who had on average more spatially precise representations (i.e., lower mean δ) scored also higher in fluid intelligence (Load 2: *r* = −0.47, *p* < 0.05, Load 3: *r* = −0.51, *p* < 0.05). For the variance components of spatial precision, the item-to-item variability component was significantly related to fluid intelligence scores for both Load 2 (*r* = −0.47, *p* < 0.05) and Load 3 conditions (*r* = −0.44, *p* < 0.05) (cf. Figure 5, first row). No significant associations were observed for the trial-to-trial (Load 2: *r* = 0.004, *p* = 0.97; Load 3: *r* = 0.19, *p* = 0.09) and occasion-to-occasion variance component (Load 2: *r* = −0.2, *p* = 0.08; Load 3: *r* = 0.09, *p* = 0.47). The day-to-day variance component showed a significant relationship for Load 2 (*r* = −0.37, *p* < 0.05) but no significant association for Load 3 (*r* = −0.03, *p* = 0.78). Thus, children’s fluid intelligence was significantly linked to both mean and variability of spatial precision. Notably, among variance components, variability from item to item showed most consistent associations with fluid intelligence, where lower variability under both loads was linked to higher fluid IQ.

Secondly, to examine convergent, divergent and predictive validity of item-to-item variability, we conducted additional correlation and hierarchical regression analyses. Results revealed a significant positive correlation between the item-to-item variability assessed on Load 2 and Load 3 conditions (*r* = 0.76, *p* < 0.05), which denotes high convergent validity of this construct. In addition, higher item-to-item variability was significantly linked to lower mean spatial precision (i.e., higher mean δ) (Load 2: *r* = 0.96, Load 3: *r* = 0.95, both *p* < 0.05) and to lower mean accuracy (Load 2: *r* = −0.95, Load 3: *r* = −0.94, both *p* < 0.05), which suggests low divergent validity between mean performance and item variability. To inspect the predictive validity of item-to-item variability of spatial precision compared to mean spatial precision on fluid intelligence, we compared three models including mean precision (Model 1), item-to-item variability (Model 2), or both mean and variability of spatial precision (Model 3) as predictor variables. We found a significant prediction of fluid intelligence by mean spatial precision (Load 2: *R*^2^ = 0.22, Load 3: *R*^2^ = 0.26, both *p* < 0.05) and item-to-item variability (Load 2: *R*^2^ = 0.22, Load 3: *R*^2^ = 0.19, both *p* < 0.05). Importantly, results demonstrated highest multiple *R*^2^ for Model 3 including both mean and variability of spatial precision (i.e., Load 2: *R*^2^ = 0.23, Load 3: *R*^2^ = 0.28, both *p* < 0.05), whereby Model 3 showed a significantly higher *R*^2^ than Model 2 for the Load 3 condition (Load 3: *F* = 9.41, *p* < 0.05). No such effect was observed for the Load 2 condition (Load 2: *F* = 0.74, *p* = 0.39), or when comparing Model 3 with Model 1 (Load 2: *F* = 0.23, *p* = 0.63; Load 3: *F* = 2.24, *p* = 0.14) (see also Figure S3 in the Supplement for a correlation matrix between mean and variability components and fluid intelligence).

Thirdly, we explored associations between children’s mean and item-to-item variability of spatial precision and measures of school achievement. Correlation analysis revealed that children with lower precision variability scored higher in a test of mathematical skills (Load 2: *r* = −0.30, Load 3: *r* = −0.35, both *p* < 0.05) and in a test of reading comprehension (Load 2: *r* = −0.54, Load 3: *r* = −0.51, both *p* < 0.05). Also, mean spatial precision was significantly correlated with both academic abilities, whereby higher precision (i.e., lower mean δ) was associated with better math (Load 2: *r* = −0.32, Load 3: *r* = −0.36, both *p* < 0.05) and better reading skills (Load 2: *r* = −0.53, Load 3: *r* = −0.53, both *p* < 0.05). Results of hierarchical regression analyses revealed significant prediction of children’s math skills by mean spatial precision (Model 1: Load 2: *R*^2^ = 0.10, *p* < 0.05; Load 3: *R*^2^ = 0.13, *p* < 0.05) as well as item-to-item variability (Model 2: Load 2: *R*^2^ = 0.09, *p* < 0.05; Load 3: *R*^2^ = 0.12, *p* < 0.05). Model 3 with both mean and variability of spatial precision as predictor variables showed a significant effect for Load 3 (*R*^2^ = 0.13, *p* < 0.05) but not for Load 2 (*R*^2^ = 0.10, *p* = 0.01). We observed no significant difference in *R*^2^ between Model 3 and Model 2 (Load 2: *F* = 1.39, *p* = 0.24, Load 3: *F* = 0.70, *p* = 0.40), or Model 3 and Model 1 (Load 2: *F* = 0.13, *p* = 0.72; Load 3: *F* = 0.05, *p* = 0.82). Also, children’s reading skills could be significantly predicted by mean spatial precision (Model 1: Load 2: *R*^2^ = 0.28, *p* < 0.05; Load 3: *R*^2^ = 0.28, *p* < 0.05) and item-to-item variability (Model 2: Load 2: *R*^2^ = 0.29, *p* < 0.05; Load 3: *R*^2^ = 0.26, *p* < 0.05). Model 3 with both mean and variability of spatial precision showed significant effects too (Load 2: *R*^2^ = 0.29, *p* < 0.05; Load 3: *R*^2^ = 0.28, *p* < 0.05). Model 3 did not differ from Model 2 (Load 2: *F* = 0.11, *p* = 0.75, Load 3: *F* = 1.91, *p* = 0.17) or from Model 1 (Load 2: *F* = 1.36, *p* = 0.25; Load 3: *F* = 0.03, *p* = 0.86).

In sum, these results suggest that variability of spatial precision is related to fluid intelligence as well as school achievement in children. In particular, children with more stable spatial precision representations from item-to-item within trials showed higher fluid intelligence and school achievement scores than children with less stable representations. The mean spatial precision component also showed a strong link to the measure of fluid intelligence and scholastic abilities. Further, the item-to-item variability construct showed high convergent validity and low divergent validity as compared to children’s mean spatial precision. For the high load condition, we observed that item-to-item variability together with mean spatial precision showed higher predictive validity for fluid intelligence than item-to-item variability alone. Thus, there is currently no indication that the item-to-item variability component is better than mean spatial precision at predicting fluid intelligence or school achievement. Note, however, that high correlations between mean spatial precision and item-to-item variability in spatial precision implicate high communality between these two variables. High communality could indicate similar or the same processes that underlie children’s mean and item-to-item variability of spatial working memory updating.

## 4. Discussion

By using cognitive ambulatory assessment, this study provides novel evidence that the spatial precision with which items are stored characterizes children’s WM performance in real-world and real-time contexts. Hierarchical modeling revealed substantial within-person changes in spatial precision at different time scales. Importantly, higher memory load increased the amount of item-to-item variability in children’s spatial precision but not any other variability component. Further, lower item-to-item variability of spatial precision was related to higher levels of education, higher fluid intelligence and higher school achievement. In sum, precise and transiently stable representations of spatial locations from moment to moment are associated with improved WM performance and thereby emphasize the importance to understand distinct components in contributing to WM updating development.

### 4.1. Spatial Precision as Continuous Quantitative Measure of Children’s Updating Performance

To better understand how children mentally present and update visuospatial information in working memory, we measured spatial precision in terms of the spatial distance between presented and reported item location during a sequential spatial WM updating task. Children showed substantial differences from trial to trial in how far in space their estimate differed from the true item location. Importantly, this was most pronounced when within trial average performance of response accuracy was low. These findings are in line with previous studies on the precision of visual WM representations in adults (e.g., [15,16]) and children [40,41,42,43,44] and support flexible resource accounts of visual WM capacity (e.g., [4,15]). In contrast to fixed capacity or ‘slot’ models [2,5,58], resource models account for the presence of internal noise in memory, which has been suggested to increase as a function of set size (i.e., the number of to-be-remembered items) (e.g., [4,15,19]). Here, WM capacity has been described as a continuous resource that can be flexibly distributed across all items in the visual scene. The more resource an item receives, the less noise is present in its representation and the more precise is the recall of that item [4,15]. Based on these assumptions, cognitive modeling studies in adults observed a critical trade-off between the number of stored items and the precision of WM representation, that is, precision declined as load on WM increased [15,16,20,21,22]. These findings are consistent with our observation that mean spatial precision substantially decreased from memory loads two to three and thereby limited children’s performance. Note that cognitive modeling studies on visual WM precision are typically based on a continuous recall paradigm which allows to measure behavioral performance (i.e., error) that is distributed along a continuous feature dimension (e.g., orientation, color) [19]. Our results are attributable to a spatial WM updating task which typically relies on a binary measure of each response, that is, correct vs. incorrect recall of item location. This task is well-established in the visual WM updating literature [37,55,59,60] but studies using fidelity measures of children’s updating performance are still missing. Inspired from studies on visual WM capacity and the continuous recall paradigm, we could show that incorrect responses during WM updating do not necessarily mean that children had no memory representation of the target locations at all. Therefore, we suggest that continuous measures of spatial precision provide additional insights in children’s response behavior during WM updating in addition to binary measures of response accuracy.

### 4.2. Systematic Variability in Children’s Spatial Precision

More recently proposed resource models of visual WM suggest that mental resource is variably but not equally distributed across items. Therefore, mnemonic precision is itself variable over time within individuals and task conditions [20,21]. Factorial model comparison revealed that this within-person variability of precision accounts for a significant proportion of errors in recall whereby variable precision models outperformed models that did not consider variability in precision [22]. Following these assumptions, we examined whether children’s spatial precision during WM updating varies over time and whether this variability depends on memory demands. Results of hierarchical modeling revealed substantial variability of spatial precision at different time scales including variation within and across days, while in particular item-to-item variability showed systematic increases from memory loads two to three. These results support the conception that specifically variability across items plays a role for variable memory precision within individuals [20,21]. Further, our findings are consistent with recent modeling results of visual WM performance impairments under high load due to increased variability of precision [20,22,61].

A growing body of evidence including our study found increased variability in cognitive performance with higher task demand or cognitive load [37,62,63,64,65]. The majority of these findings are based on developmental or lifespan research on variability of trial-to-trial reaction time (RT) measures. Here, we combined intensive longitudinal assessment of cognitive performance and hierarchical modeling which allowed us to directly test which time scales are most important for performance limitations. We identified that it is indeed the fast item-to-item variability which was increased with higher memory load and thereby affects performance limitations. Variability from item to item has been recently reported by measuring memory performance for all items in each trial of a continuous recall task and thereby claiming that guesses, not low-precision representations, determines visual WM limitations [66]. Here, we do not want to exclude the possibility that some portion of the incorrect responses were merely random guesses with a uniform distribution across all fields of the grid. However, the goal of the present study was to identify how variability in children’s item-responses changes at different time scales, rather than to separate guessing from precision (for which a higher number of observations within a broader feature space would be necessary). Beyond this ‘slot’ vs. resource debate on WM capacity (for reviews see [3,67]), neurocognitive studies have proposed different potential mechanisms that may underlie variability in cognitive performance [23,24,61,68,69]. Possible sources of variability in spatial precision may result from internal process-related fluctuations (e.g., sleep quality based on circadian functions, cf. [38]) but also external factors such as environmental noise (cf. [68]). Variability in dopaminergic activity in prefrontal cortex was found to modulate visuospatial WM performance [24,25,26] and thus, may reflect a potential neural source underlying variability in spatial precision. The high correlation between mean performance and variability in performance suggests that similar or even the same processes may underlie the two components, while it will become necessary to use further experimental manipulations and/or neuroimaging methods to convincingly identify this proposed communality of underlying processes. Based on previous findings on visual WM precision, we speculate that attentional mechanisms constitute an important source underlying item-to-item variability of spatial precision [15,21,23,69]. One possibility is that such variation may result from random fluctuations in attention and that these fluctuations increase when multiple locations have to be processed, that is encoded, stored and updated and finally recalled. Another potential mechanism may be less controlled shifts in attention when demands on WM are high, while more controlled allocation of selective attention may stabilize WM performance and thereby improve spatial precision [15,23].

### 4.3. Individual Differences and Developmental Changes in Variability of Spatial Precision

Combining the concepts of short-term within-person variability such as performance variations across and within days and theories of long-term change during development has been proven to be a worthwhile concept of understanding individual dynamics in cognitive functions [31,70]. Following these calls, we attempted to measure short-term within-person variability in WM performance over a period of four weeks in third as well as fourth graders. Further, we measured children’s performance with smartphones in typical settings, such as in school and after school to increase ecological validity [32,71]. We observed that third graders with mean age of approximately nine years showed higher item-to-item variability of spatial precision of WM performance compared to the around ten-year-old fourth graders. These results fall in line with previous developmental research on within-person variability of RT measures (i.e., SD of RTs) and variability in accuracy at faster time scales, which together found a reduction in performance variability with increasing age during childhood [37,65,72]. Moreover, not only younger but also older populations [72,73,74] and patients with attention-deficit hyperactivity disorder (ADHD) showed increased trial-to-trial RT variability [75,76], which has been suggested to reflect reduced resolution of information processing systems [73,74]. Our findings refine these results and point to the importance of transient within-person changes in spatial WM precision for long-term changes during development of educational competencies.

In addition to reduced item-to-item variability, we observed that Grade 4 students were on average more precise in spatial recall than Grade 3 students. This finding fits well to the results of recent developmental studies on age-related changes in visual WM capacity using the continuous recall paradigm [40,41,42,43]. These studies found reduced errors in recall over middle childhood development, while they came up with mixed conclusions whether this performance improvement is due to increases in WM precision [40,41] or reduced guessing behavior [42]. Developmental improvements in WM resolution have been observed already in younger children (i.e., between four and six years old) in experimental manipulations of the precision of colors within a color discrimination paradigm [44]. These as well as our findings support assumptions of the dynamic field theory which predicts that neuronal interactions in visuospatial WM become more spatially precise over development, resulting in more stable behavior (i.e., spatial precision hypothesis; [44,77]).

The observed grade differences in spatial precision performance may be associated with differences between children in their fluid intelligence and their school achievement [37]. More mature self-regulatory processes with increased age and level of education may also explain grade differences [37,78]. Further, we observed strong associations between mean and variability of spatial precision and fluid intelligence as well as school achievement. In particular, children with higher mean spatial precision and lower item-to-item variability showed higher fluid intelligence and higher math and reading abilities. These findings support and extend previous results on WM in predicting higher-level abilities such as learning and intelligence [11,12,13,14].

### 4.4. Future Perspectives and Limitations

The present study extends existing research in important ways by showing that children’s spatial WM is not stable over time but substantially varies across days, occasions, trials and items. Specifically, the item-to-item variability systematically changes with memory load and level of education, thereby reflecting a new index of performance limitations in children’s everyday life. It is however important to note that, in contrast to previous research on the variability of WM precision [20,21], we worked with a spatial WM updating task that is inherently different and more complex than the continuous recall paradigm [19]. To mentally shift multiple locations held in WM may reflect different cognitive functions than to briefly store visual features in WM, thus a direct comparison to previous cognitive modeling research using the variable precision model is restricted. To further test the assumptions of the variable precision model in children and their natural contexts, future studies could combine a continuous recall paradigm and ambulatory assessment which would allow to estimate and to compare variability at different time scales. In addition, it is important to note that with the current design we cannot distinguish whether the observed grade- (and age-)related differences in WM performance are due to effects of schooling, maturity, and/or other time-related variables. To fully understand the development of distinct components of WM capacity and to which extent WM improvements are driven by education versus maturation, further research is needed. As a future perspective, longitudinal methods and a broader age range could help to clearly separate the variance of WM components that is linked to these variables.

Moreover, whereas limits in attention reflect reasonable mechanisms of item-to-item variability in spatial precision (e.g., [21,23]), we cannot test these assumptions within the current study. Further work should focus on disentangling the mechanisms underlying variability of spatial precision and thereby limitations in children’s updating performance. Variability in spatial precision from item to item may result from early perceptual and attentional limitations during encoding but could also stem from constraints in memorizing, mentally shifting, and/or retrieving information in WM. Thus, future studies should examine the specific WM sub-processes and how their interaction affects variability in spatial precision. For example, to better understand updating-related processes, one could measure children’s estimates of remembered locations after each updating step by estimating spatial precision and its variability across updates. Further, technical advances in combining ambulatory assessment and neurocognitive methods such as mobile electroencephalography (EEG) (e.g., [79]) may reflect a fruitful approach to relate neural correlates of WM sub-processes to children’s behavioral performance in real-life contexts.

In addition to variability from item to item, spatial precision showed substantial fluctuations also at slower time scales such as days and occasions within days. These fluctuations were independent of memory load and school grade, suggesting a less detrimental effect of these more enduring within-person changes for performance limitations. However, children considerably differed in their amount of day-to-day or occasion-to-occasion variability and also in whether they showed an increase, decrease or no change in these variabilities with load. To better understand these individual differences, the relationships of spatial precision variability at slower time scales with other daily varying constructs may shed light on some influential factors [37], such as sleep [38] or physical activity [51].

The combination of ambulatory assessment and hierarchical modeling allowed us to provide improved knowledge about short-term changes in children’s behavior in everyday life settings. This may be specifically important for developmental and lifespan research, as cognitive development is a dynamic process which is not constrained to laboratory settings [32,71]. Following these calls, we were able to assess children’s WM performance at different time scales with high density of observations in their natural contexts. Aside from these important aspects of ambulatory assessment, there are methodological constraints, for example regarding the compliance with and reactivity to study procedures during data collection [71]. Thus, an important future perspective is the improvement of such aspects, for example, by implementing reward systems within intensive longitudinal designs to enhance children’s study motivation.

## Supplementary Materials

The following are available online at http://www.mdpi.com/2079-3200/6/1/8/s1, Table S1: Summary statistics of daily measures of spatial precision in updating, Table S2: Descriptive statistics of spatial precision variance components at different time scales.
