# Supplementary material for: Variability in the Precision of Children’s Spatial Working Memory
Source: J Intell. 2018 Feb 28;6(1):8. doi: 10.3390/jintelligence6010008 (PMC6480713; doi:10.3390/jintelligence6010008)
Supplement: Supplementary file 1 [file jintelligence-06-00008-s001.pdf]

# Supplementary Materials for

# Variability in the precision of children's spatial working memory

**Table S1.** Summary statistics of daily measures of spatial precision in updating.

| Grade | Load | Occasion | *M* | *SD* | *M (ISD)* | *SD (ISD)* |
|---|---|---|---|---|---|---|
| 3 | 2 | Morning | 0.58 | 0.43 | 0.78 | 0.27 |
| | | Noon | 0.76 | 0.42 | 0.89 | 0.22 |
| | | Afternoon | 0.63 | 0.44 | 0.78 | 0.26 |
| | 3 | Morning | 0.94 | 0.41 | 0.92 | 0.13 |
| | | Noon | 1.11 | 0.39 | 0.97 | 0.10 |
| | | Afternoon | 1.01 | 0.41 | 0.94 | 0.12 |
| 4 | 2 | Morning | 0.30 | 0.23 | 0.65 | 0.21 |
| | | Noon | 0.43 | 0.30 | 0.76 | 0.24 |
| | | Afternoon | 0.38 | 0.27 | 0.70 | 0.25 |
| | 3 | Morning | 0.67 | 0.35 | 0.86 | 0.16 |
| | | Noon | 0.83 | 0.36 | 0.93 | 0.15 |
| | | Afternoon | 0.76 | 0.36 | 0.90 | 0.19 |

**M* = mean, *SD* = standard deviation, *ISD* = intraindividual *SD*, n=110

**Table S2.** Descriptive statistics of spatial precision variance components at different time scales.

| Grade | Load | Time scale | *M* | *SD* | *Median* | *Min* | *Max* |
|---|---|---|---|---|---|---|---|
| 3 | 2 | Day | 0.037 | 0.058 | 0.001 | 0.000 | 0.192 |
| | | Occasion | 0.042 | 0.032 | 0.038 | 0.000 | 0.121 |
| | | Trial | 0.023 | 0.009 | 0.020 | 0.001 | 0.047 |
| | | Item | 0.011 | 0.007 | 0.010 | 0.001 | 0.028 |
| | | Total | 0.112 | 0.076 | 0.107 | 0.002 | 0.311 |
| | 3 | Day | 0.023 | 0.033 | 0.002 | 0.000 | 0.130 |
| | | Occasion | 0.030 | 0.024 | 0.030 | 0.000 | 0.076 |
| | | Trial | 0.020 | 0.008 | 0.018 | 0.006 | 0.036 |
| | | Item | 0.014 | 0.005 | 0.014 | 0.005 | 0.024 |
| | | Total | 0.087 | 0.046 | 0.085 | 0.019 | 0.219 |
| 4 | 2 | Day | 0.021 | 0.042 | 0.000 | 0.000 | 0.216 |
| | | Occasion | 0.035 | 0.043 | 0.016 | 0.000 | 0.202 |
| | | Trial | 0.023 | 0.012 | 0.026 | 0.003 | 0.046 |
| | | Item | 0.005 | 0.004 | 0.004 | 0.000 | 0.021 |
| | | Total | 0.083 | 0.065 | 0.070 | 0.003 | 0.251 |
| | 3 | Day | 0.023 | 0.036 | 0.007 | 0.000 | 0.157 |
| | | Occasion | 0.029 | 0.027 | 0.022 | 0.000 | 0.130 |
| | | Trial | 0.023 | 0.008 | 0.024 | 0.006 | 0.039 |
| | | Item | 0.012 | 0.004 | 0.013 | 0.002 | 0.019 |
| | | Total | 0.086 | 0.048 | 0.079 | 0.019 | 0.251 |

[1] *M* = mean, *SD* = standard deviation, n=34 (Grade 3), n= 49 (Grade 4).

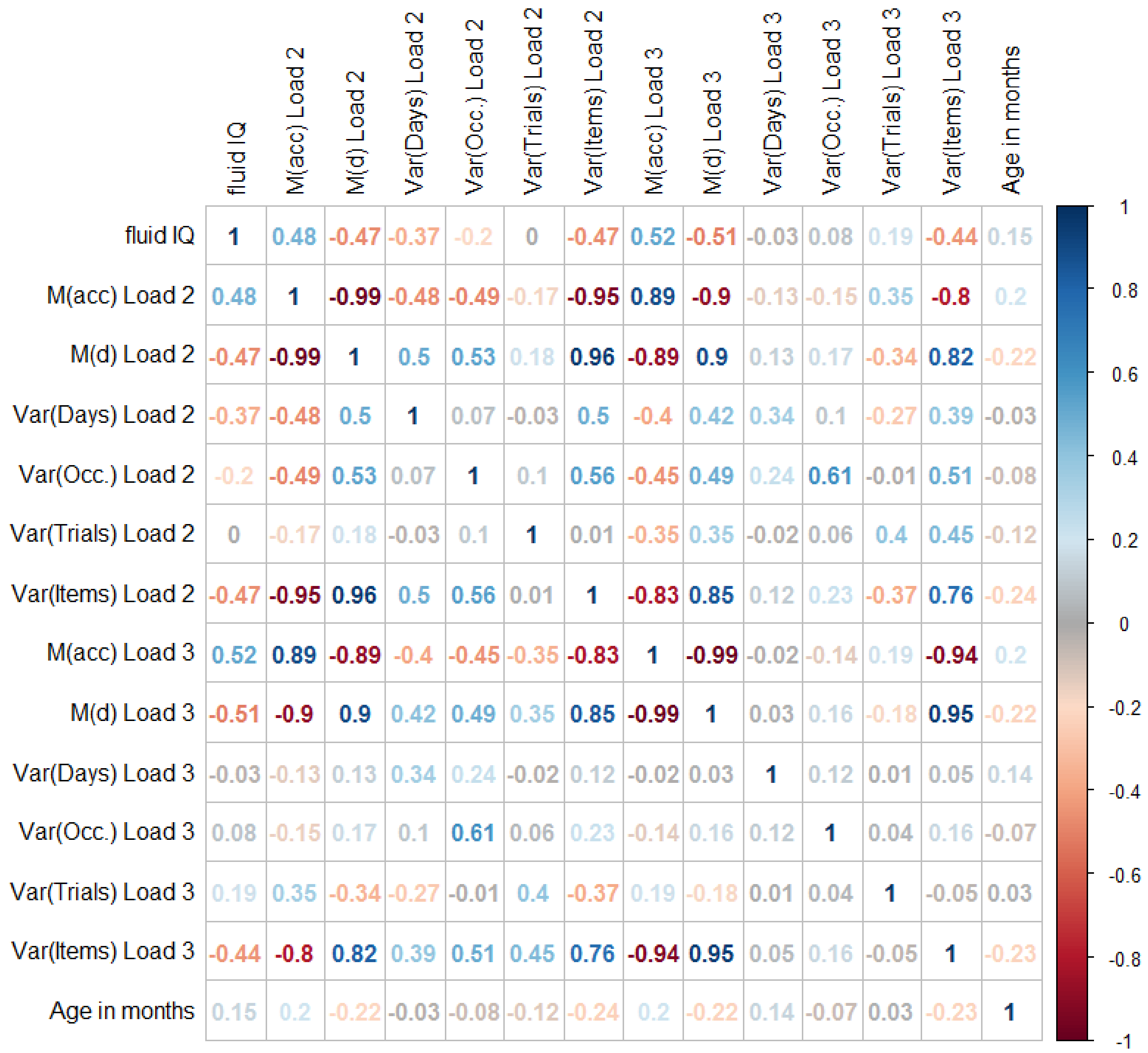


**Figure S3.** Correlation matrix with Pearson correlation coefficients of working memory updating components (mean accuracy, mean spatial precision, and variability of spatial precision) for the two load conditions (i.e.. Load 2 and 3), fluid intelligence, and age (n = 82). *M* = mean, *Var* = variance component, acc = accuracy, *d* = spatial precision, Occ. = occasion.
